# Differential requirements of protein geranylgeranylation for the virulence of human pathogenic fungi

**DOI:** 10.1080/21505594.2019.1620063

**Published:** 2019-05-25

**Authors:** Ana Camila Oliveira Souza, Qusai Al Abdallah, Kaci DeJarnette, Adela Martin-Vicente, Ashley V. Nywening, Christian DeJarnette, Emily A. Sansevere, Wenbo Ge, Glen E. Palmer, Jarrod R Fortwendel

**Affiliations:** a Department of Clinical Pharmacy and Translational Science, College of Pharmacy, University of Tennessee Health Science Center, Memphis, TN, USA; b Department of Molecular Immunology and Biochemistry, College of Graduate Health Sciences, University of Tennessee Health Science Center, Memphis, TN, USA

**Keywords:** Aspergillus fumigatus, Candida albicans, prenylation, prenyltransferases, geranylgeranylation, geranylgeranyltransferase-I, protein localization, fungal virulence

## Abstract

Protein prenylation is a crucial post-translational modification largely mediated by two heterodimeric enzyme complexes, farnesyltransferase and geranylgeranyltransferase type-I (GGTase-I), each composed of a shared α-subunit and a unique β-subunit. GGTase-I enzymes are validated drug targets that contribute to virulence in *Cryptococcus neoformans* and to the yeast-to-hyphal transition in *Candida albicans*. Therefore, we sought to investigate the importance of the α-subunit, RamB, and the β-subunit, Cdc43, of the *A. fumigatus* GGTase-I complex to hyphal growth and virulence. Deletion of *cdc43* resulted in impaired hyphal morphogenesis and thermo-sensitivity, which was exacerbated during growth in rich media. The Δ*cdc43* mutant also displayed hypersensitivity to cell wall stress agents and to cell wall synthesis inhibitors, suggesting alterations of cell wall biosynthesis or stress signaling. In support of this, analyses of cell wall content revealed decreased amounts of β-glucan in the Δ*cdc43* strain. Despite strong *in vitro* phenotypes, the Δ*cdc43* mutant was fully virulent in two models of murine invasive aspergillosis, similar to the control strain. We further found that a strain expressing the α-subunit gene, *ramB*, from a tetracycline-inducible promoter was inviable under non-inducing *in vitro* growth conditions and was virtually avirulent in both mouse models. Lastly, virulence studies using *C. albicans* strains with tetracycline-repressible *RAM2* or *CDC43* expression revealed reduced pathogenicity associated with downregulation of either gene in a murine model of disseminated infection. Together, these findings indicate a differential requirement for protein geranylgeranylation for fungal virulence, and further inform the selection of specific prenyltransferases as promising antifungal drug targets for each pathogen.

## Introduction

Protein prenylation is a post-translational modification that increases protein hydrophobicity, promoting protein–protein interactions and protein affinity for cell membranes [1]. This modification is critical for the subcellular localization and function of important proteins, being crucial in various cellular physiological processes. The reaction is mediated by prenyltransferases (PTases) and consists of the transfer of an isoprenoid lipid, derived either from a 15-carbon farnesyl pyrophosphate (FPP) or a 20-carbon geranylgeranyl pyrophosphate (GGPP), to proteins that bear conserved cysteine residues. Three types of prenyltransferases have been identified in eukaryotes: Farnesyltransferase (FTase), Geranylgeranyltransferase type-I (GGTase-I) and type-II (GGTase-II). FTase and GGTase-I similarly recognize a consensus sequence of four amino acids that is usually referred to as the CaaX motif (C = cysteine; aa = two aliphatic amino acids, usually; X = any amino acid) [1–3]. In contrast, protein substrates that are prenylated by GGTase-II harbor CXC or CC recognition motifs [1–3]. Recently, it has been demonstrated that proteins bearing a C-terminus CaaaX sequence may also be subject to prenylation by Ftase enzymes [4].

The CaaX prenyltransferases, GGtase-I and FTase, are conserved heterodimeric zinc metalloenzymes with a common α- subunit and unique β-subunits [5]. The active site is located at the interface of the two domains, and the catalytic reaction consists of the covalent attachment of the isoprenyl group to the sulfur atom of the cysteine residue in the CaaX motif [2,6]. The last amino acid in the CaaX motif partially dictates substrate specificity of the PTase reaction. Classically, GGTases prenylate CaaX motifs in which X is a leucine or isoleucine, whereas substrate proteins ending in a glutamine, methionine, serine, cysteine or alanine are farnesylated [1,7,8]. However, cross-specificity has been reported [9]. After prenylation, the substrate protein can be further processed by proteolysis of the aaX residues and carboxy-methylation [1,10].

Among the important protein substrates that bear a CaaX motif are small GTPases, such as the Ras- and Rho-like proteins, pheromones, lamins and trimeric G-proteins [1,11]. In mammalian cells, these proteins play crucial roles in cell-signaling pathways and are involved in human conditions such as cancer, inflammatory and aging disorders and neurodegenerative diseases [3]. In fungal organisms, knowledge of the protein prenylome is still relatively limited and concentrated on the Ras- and Rho-family proteins. These proteins are involved in fungal growth, morphogenesis, and pathogenesis [12,13]. The absence of prenylation potentially culminates in protein mislocalization, which in turn can drastically impair protein function and have pleiotropic consequences on cellular homeostasis. For this reason, prenylation pathways have been explored as drug targets to limit tumor growth, via inhibition of oncogenic Ras signaling in humans, and to battle infectious diseases, in particular, fungal and parasite infections [6,14–18]. Prenyltransferases are validated targets in mammals and fungi, and several inhibitors were identified and are currently under investigation [3,16,19,20]. Some of these inhibitors were found to be active against fungal enzymes or cells [18,21–24]. Importantly, it has been reported that differences in the tertiary structure of the substrate binding pocket of mammalian versus fungal prenyltransferases may allow the design of inhibitors that could specifically target fungal enzymes, avoiding potential cytotoxicity in host cells [6,24,25].

Protein prenyltransferases have been extensively characterized in fungi, especially yeast organisms. The shared α-subunit of fungal PTases, encoded by *RAM2*, is essential for the viability of the yeasts *Saccharomyces cerevisiae* [26], *Candida albicans* [21] and *C. glabrata* [27]. The β-subunit of FTase (in yeasts encoded by *RAM1* or *cpp1^+^*) was found to mediate morphogenesis in *Cryptococcus neoformans* [28] and *Schizosaccharomyces pombe* [29]. *S. cerevisiae* [26,30] and *C. neoformans* [28] *RAM1* mutants also display thermosensitive growth at 37°C. Accordingly, deletion of *RAM1* attenuates *C. neoformans* pathogenicity in a murine model of cryptococcosis [28]. *CDC43*, which is the β-subunit of GGTase-I, is essential in *S. cerevisiae* [26], and mediates morphogenesis in *C. albicans* [31] and thermotolerance, morphology, and virulence in *C. neoformans* [32]. Recently, we characterized the β-subunit of the farnesyltransferase complex (RamA) in *Aspergillus fumigatus* and demonstrated that *ramA* null mutants display attenuated virulence, accompanied by growth defects and decreased conidial viability [33]. In this mutant, localization of RasA was only partially impaired, suggesting that lack of Ras farnesylation could be compensated by GGTase-I activity. However, the role of protein geranylgeranylation has not yet been accessed in filamentous fungi.

Therefore, in this work, we sought to identify and study the β-subunit of GGTase-I (Cdc43) and the shared α-subunit (RamB) in *A. fumigatus*. We observed that *A. fumigatus cdc43* mutants displayed a thermosensitivity phenotype, slow growth, abnormal hyphal morphogenesis, and a defective cell wall. Despite *in vitro* phenotypes, the Δ*cdc43* mutant was fully virulent in two models of murine invasive aspergillosis. We also demonstrate that the expression of the α-subunit, RamB, is essential for *A. fumigatus* viability *in vitro* and for invasive growth in mouse models of invasive aspergillosis. Lastly, we found that both *CDC43* and *RAM2* expression are essential for *C. albicans* pathogenicity in a murine model of invasive candidiasis. Taken into context with our previous reports examining the importance of farnesyltransferase activity in *A. fumigatus*, our findings here reveal differential requirements for specific prenyltransferases during growth, morphogenesis, and virulence. Further exploration of the roles of the virulence-associated prenylome will identify individual substrate proteins underlying phenotypes associated with farnesyltransferase and geranylgeranyltransferase inhibition, identifying novel factors driving the pathogenesis of invasive aspergillosis.

## Materials and methods

### Genetic manipulation of *A. fumigatus*

All strains utilized in this study are listed in Table 1, and oligonucleotides designed for strain construction are listed in Table 2. The generation and transformation of *A. fumigatus* protoplasts were performed using a previously described protocol [34]. The *A. fumigatus* genetically engineered KU80∆pyrG strain (Δ*akuB*) [35] was used as a background for all strains developed in this study. This strain exhibits high rates of homologous recombination due to its deficiency in the non-homologous end joining (NHEJ) DNA repair pathway and is also uracil auxotrophic (*pyrG-*). The control strain ∆*akuB-pyrG*^+^ (Ctrl) was obtained previously [36] by replacing the non-functional *pyrG* locus of the *A. fumigatus* KU80∆pyrG strain [35] with the functional homolog from *A. parasiticus* obtained from plasmid pJW24 [37]. The *cdc43* deletion mutant (Δ*cdc43*) was obtained by replacement of the entire ∆*akuB cdc43* coding sequence using the *A. parasiticus pyrG* gene, as previously described [36]. Briefly, 1.5 kb fragments of genomic sequence located immediately upstream and downstream of the putative *cdc43* coding locus were first PCR amplified using primer sets P1/P2 and P3/P4. These primers incorporated restriction enzyme sites utilized for sub-cloning of each fragment as flanking arms of the *pyrG* cassette in vector pJW24. The final deletion cassette was PCR amplified from the resulting vector using the primer set P1/P4 and utilized for transformation (Supp Figure 1). Complementation of Δ*cdc43* (Δ*cdc43::cdc43*) was achieved by the ectopic integration of the entire *cdc43* coding sequence, including 1 kb of upstream and 300 bp of downstream genomic sequence. This fragment was PCR amplified from genomic DNA using primer set P5/P6 and was cloned into a *NotI* restriction site immediately upstream of a hygromycin resistance cassette in vector pHygR [38]. Primers P5/P7 were then used to amplify the full *cdc43* coding locus and downstream *hygR* cassette for ectopic integration into the Δ*cdc43* mutant (Supp Figure 1). The *ramB* (pTetOn-*ramB*) tetracycline-inducible strain was developed by replacement of the native *ramB* promoter with the pyrithiamine-based Tet-On cassette from plasmid pCH008 [39] using an overlap extension PCR method. In short, a 1.5 kb fragment of the upstream genomic sequence was first amplified from genomic DNA using primer set P8/P9. In a second reaction, 1.5 kb of the sequence starting at the putative *ramB* start codon and stretching downstream was PCR amplified from genomic DNA with primer set P10/P11. In a third reaction, the entire pyrithiamine-Tet-On construct was PCR amplified from vector pCH008 using primers P12/P13. Finally, the three resulting fragments from the above reactions were utilized in a single overlap extension PCR reaction using nested primers P14/P15 and the full construct was utilized for transformation (Supp Figure 1). To ensure the expression of the *ramB* gene, the mutant was maintained on doxycycline (30 µg/ml)-impregnated growth media. All the strains were confirmed by PCR and Southern blot analyses.

**Table 1.** A. fumigatus strains utilized in this study.

Strain name	Background
∆*akuB-pyrG*^+^36	KU80∆pyrG strain35
Δ*cdc43*	∆*akuB-pyrG*^+^36
Δ*cdc43::cdc43*	Δ*cdc43*
pTetOn-*ramB*	∆*akuB-pyrG*^+^ 36

**Table 2.** Oligonucleotides used for genetic manipulation of A.fumigatus.

Primer	Sequence	Purpose
P1	TTTTGCGGCCGCCAGGCTGAACGGTTTATGGG	Subcloning of 1.5kb upstream flanking region of *cdc43* in pJW24; amplification of *cdc43* deletion cassette
P2	TTTTGCGGCCGCTGACAATGTTGAATCTGCTCG	Subcloning of 1.5kb upstream flanking region of *cdc43* in pJW24
P3	TTTTCTCGAGTGCTCTAAATTATAGTGGACGGG	Subcloning of 1.5kb downstream flanking region of *cdc43* in pJW24
P4	TTTTGGTACCTGGTCAAGAAAG GAGAAGAAGC	Subcloning of 1.5kb downstream flanking region of *cdc43* in pJW24; amplification of *cdc43* deletion cassette
P5	TTTTGCGGCCGCGCACAACTCAATAAAGGCTTCCAC	Subcloning of *cdc43* coding sequence in pHygR; amplification of *cdc43* complementation cassette
P6	TTTTGCGGCCGCTAATAGTGGTCTGGTGGATGGCTTG	Subcloning of *cdc43* coding sequence in pHygR
P7	CGAGCTCCCAAATCTGTCCAG	Amplification of *cdc43* complementation cassette
P8	CACACAAGACACCTCCGCC	Amplification of the 1.5 kb upstream flanking region of *ramB*
P9	CAGCGCGAGTGTGCTGAGTAAGCTGGGACGGAATAATGATTGGTG	Amplification of the 1.5 kb upstream flanking region of *ramB*
P10	CCCGCTTGAGCAGACATCACATGGAAGGGAAATACTCGTCCGATC	Amplification of the 1.5 kb region of *ramB* from the start codon
P11	ATCCAAATCCGTACCGTTTGA ATATCC	Amplification of the 1.5 kb region of *ramB* from the start codon
P12	TTACTCAGCACACTCGCGCTG	Amplification of the Pyrithiamine-Tet-On cassette from pCH008
P13	GTGATGTCTGCTCAAGCGGG	Amplification of the Pyrithiamine-Tet-On cassette from pCH008
P14	CCAACGGGACCTGTGGC	Overlap extension PCR for *ramB* promoter replacement cassette
P15	GCATAAATGACCTCGCTCCAGC	Overlap extension PCR for *ramB* promoter replacement cassette

### *C. albicans* strains

The *C. albicans* strains with tetracycline repressible expression of either *CDC43* or *RAM2*, were kindly provided by Merck Sharp & Dohme Corp. Their construction has been previously described [40].

### Growth conditions and analyses

Strains were cultured in agar Glucose Minimum Media (GMM) [41] for a minimum of 3 days at 37°C and conidia were harvested in sterile distilled water, filtered in miracloth, washed at least twice, and concentration was determined using a hemocytometer. Where indicated, Yeast Peptone Dextrose (YPD, with 1% yeast extract, 2% peptone, 2% dextrose) and Potato Dextrose Agar (PDA) were utilized. Because the Δ*cdc43* mutants displayed decreased ability to produce conidia, except for the radial growth and cell wall stress analysis with CFW and CR, this strain was inoculated onto GMM with ammonium tartrate as the nitrogen source, instead of Na_2_NO_3,_ and incubated for at least 5 days at 30°C.

Conidia (5x10^3^) from each strain were point inoculated onto 60 mm diameter agar plates containing the indicated media and incubated at the designated temperatures (27°C to 45°C). Colony diameter was measured every 24 h over 96 total hours of incubation at 37°C. Experiments were performed three times, using conidia derived from independent cultures, and statistical analyses were done using two-way ANOVA with Tukey’s test for multiple comparison (GraphPad Prism 7.00). For growth analysis of the pTetOn-*ramB* strain, 6-well plates containing GMM agar supplemented with a range of doxycycline concentrations (1, 5, 10, 20 and 30 µg/ml) were point inoculated with conidia (5x10^3^) and incubated for 3 days at 37°C. For analysis of micromorphology at 30°C and 37°C, conidia (10^5^) were inoculated on sterile coverslips submerged in GMM broth (3 ml) in six-well plates. After the designated time points, coverslips were mounted onto slides and visualized by bright field microscopy. Germination rates were determined after 5, 7, 10, 12, 15, and 24 h of incubation. At least 100 conidia were examined, and the percentage of conidia that produced a visible germ tube was determined at each time point. Analyses were performed six times using conidia derived from independent cultures. Statistical differences were analyzed using two-way ANOVA with Tukey’s test for multiple comparison (GraphPad Prism 7.00).

All *C. albicans* strains were routinely grown in YPD broth or on YPD agar plates at 30°C, unless otherwise stated.

### Cell wall stress analysis

Growth in the presence of cell wall stressing agents was evaluated by inoculating 10-fold serial dilutions ranging from 10^2^ to 10^5^ conidia (5 µl) onto GMM agar containing Congo Red (CR) at 20 µg/ml or Calcofluor White (CFW) at 150 µg/ml. Colonies were examined after 48-h incubation at 37°C. Susceptibility to chitin and β-glucan biosynthesis inhibition was assessed through a broth microdilution assay with Nikkomycin Z and Caspofungin, respectively, following the guidelines of the document M38-A2 of CLSI [42] with adaptations. For chitin biosynthesis inhibition, conidia (2x10^4^) were inoculated into GMM broth with ascending doses of the Nikkomyzin Z (ranging from 0.1 to 100 µg/ml) and incubated for 24 h at 37°C. For β-glucan biosynthesis inhibition, conidia (4x10^4^) were inoculated into RPMI with ascending doses of Caspofungin (ranging from 8 to 0.01 µg/ml) and incubated for 24 h at 35°C. Minimum inhibitory concentration (MIC) of Nikkomycin Z, with complete inhibition of growth, and Minimum effective concentration (MEC) of Caspofungin, corresponding to the lowest concentration leading to abnormal hyphal growth, were determined after microscopic visualization.

### Analysis of cell wall β-glucan content

For visualization of β-glucan in the cell wall by fluorescence microscopy, conidia (2x10^7^) were grown in filtered GMM broth (10 ml) and incubated at 30°C or 37°C in a rotatory shaker (250rpm). When germ tubes were visible, germlings were harvested by centrifugation, washed in sterile PBS twice, and incubated in a PBS solution with 10 µg/ml of Fc-hDectin-1a (Invivogen), which is an engineered soluble human Dectin-1 receptor fused with a human IgG1 Fc domain. Fc-hDectin-1a binds to β-glucan residues through the Dectin-1 domain, and also allows binding of a tagged secondary antibody to the IgG1 Fc-domain. After 1 h of incubation with Fc-hDectin-1a at 4°C, samples were washed twice with PBS and incubated with a 1:200 dilution of a FITC-conjugated anti-human-IgGFc (Thermofisher). Samples were once again incubated for 1 h at 4°C, washed twice with PBS and mounted for visualization of β-glucan using the GFP filter in a Nikon NiU microscope equipped with a Nikon DS-Qi1Mc camera. Images were captured using Nikon Elements software (v4.0).

Quantification of total β-glucan was performed as previously described [41,43]. Briefly, conidia (2x10^8^) were inoculated into 100 ml of GMM broth and allowed to grow overnight at 27°C, 30°C, or 37°C. Hyphae were harvested by filtration with miracloth, washed in 0.1 M NaOH, and lyophilized for 16 h. Dry weight was recorded and samples were disrupted in a bead-beater for 3x 1-min cycles each followed by 1 min on ice. The resulting powder was resuspended in 1 M NaOH for a final concentration of 20 mg/ml and incubated at 52°C for 30 min. Fifty-microliter triplicates from each sample were aliquoted into a masked, 96-well fluorescence plate and mixed with 185 µl of aniline blue staining solution (183 mM glycine, 229 mM NaOH, 130 mM HCl, and 618 mg/l aniline blue, pH 9.9, incubated overnight in the dark to decolorization). The samples were then incubated for an additional 30 min at 52°C, and allowed to cool down at room temperature for 30 min. Fluorescence readings were performed at 405 nm excitation and 460 nm emission, and a standard curve (twofold dilutions ranging from 160 µg/ml to 2.5 µg/ml) with Curdlan, a β-1,3-glucan analog, was used in order to normalize values. Experiments were performed using samples derived from three independent mycelia cultures, and statistical differences were analyzed using two-way ANOVA with Tukey’s test for multiple comparison (GraphPad Prism 7.00).

### Quantification of cell wall total chitin

Total chitin quantification was also performed as described by Fortwendel et. al. (2009) with adaptations [41]. Lyophilized hyphae were weighed, resuspended in 3 ml of 1 M KOH, and autoclaved for 60 min at 121°C. Samples were cooled to room temperature, 8 ml of ice-cold 75% ethanol were added and the resulting suspension was vortexed until a single phase was obtained. After incubation on ice for 15 min, 300 µl of a 13.3% (wt/vol) Celite 545 (C212; Fisher Chemicals) suspension was added and the tubes were centrifuged at 1,500 x g for 5 min at 2°C. The precipitates were washed with 10 ml of ice-cold 40% ethanol, followed by two washes in 10 ml ice-cold water. The final pellet from each sample was resuspended in equal volumes (500 µl) of water, 5% (wt/vol) NaNO_2_, and of 5% (wt/vol) KHSO_4_. A standard curve with glucosamine (twofold serial dilutions ranging from 100 µg/ml to 6.25 µg/ml) in water was also treated with equal volumes of 5% NaNO_2_ and 5% KHSO_4_. Tubes were mixed three times for a 15-min period, followed by centrifugation at 1,500 x g for 2 min at 2°C. Aliquots (600 µl) were mixed with 0.2 ml of 12.5% (wt/vol) NH_4_ sulfamate and mixed vigorously each minute for 5 min, followed by the addition of 0.2 ml of 3-methylbenzthiazolin-one-2-hydrazone (5 mg/ml) and incubation at 130°C for 3 min. Tubes were allowed to cool, and 0.2 ml of 0.83% (wt/vol) ferric chloride was added, followed by incubation at room temperature for 25 min. Absorbance readings were performed at 650 nm and glucosamine standard curve values were used in order to normalize values. Chitin levels were reported as glucosamine equivalents per mg of hyphae. Experiments were performed using samples derived from three independent mycelia cultures, and statistical differences were analyzed using unpaired t-test (GraphPad Prism 7.00).

### Virulence studies

All animal experiments were done according to protocols approved by the University of Tennessee Institutional Animal Care and Use Committee. For the virulence studies using *A. fumigatus* strains, six-week-old female CF-1 mice (Harlan Laboratories) were immunosuppressed using two different protocols previously described [33]. For both the non-neutropenic and neutropenic models of invasive aspergillosis, mice were subcutaneously injected with 40 mg/kg of triamcinolone acetonide one day prior to infection. For the neutropenic model, mice were additionally administered intraperitoneal injections with 150 mg/kg of cyclophosphamide 3 days prior infection and again on every third day thereafter for a total of 3 to 5 injections, depending on the health of the animal. On Day 0, mice were transiently anesthetized with 3.5% isoflurane and intranasal inoculation was performed with 10^5^ freshly harvested conidia in 20 µl of pyrogen-free saline solution. Mortality was monitored for 13–15 days, depending on the health of the animals. Survival data were plotted on a Kaplan–Meier curve and analyzed by Log-rank (Mantel-Cox) test using GraphPad Prism 7.00. For histology analysis, mice were immunosuppressed as above, inoculated with conidia and euthanized 3 days after infection. Lungs were inflated with 10% buffered formalin solution and fragments of superior, middle and inferior right lobes were further processed for paraffin-embedding. Slides containing 5 μm sections of lung tissue were stained with hematoxylin and eosin and Grocott’s methenamine silver (GMS). The presence of fungal hyphae was assessed by light microscopy.

The mouse model of hematogenously disseminated candidiasis was performed as follows. Groups of 6–8-week-old female BALB/c mice (Charles River Laboratories) were randomly assigned to one of two treatment groups three days before infection. Group 1 (-Dox) was provided 20 g of DietGel food per day, per cage of 4 mice, while group 2 (+Dox) was provided the same diet supplemented with 2 mg/ml of doxycycline hyclate. Treatment was maintained daily for the duration of the experiment (up to 16 days post-infection). Each *C. albicans* strain was grown overnight in YPD cultures at 30°C (200 rpm). Cells were washed twice in sterile endotoxin-free phosphate-buffered saline (PBS) and cell density determined using a hemocytometer. Each strain was then diluted to 5 × 10^6^ cells/ml in sterile PBS, and 0.1 ml of each cell suspension inoculated into the lateral tail vein of mice from each treatment group. Viable cell counts of each inoculum were confirmed by plating appropriate dilutions onto YPD agar plates and counting the number of colonies formed after 48 h. Mice were monitored for 16 days and those showing distress euthanized. Survival data were plotted on a Kaplan–Meier curve and analyzed by Log-rank (Mantel-Cox) test using GraphPad Prism 7.00.

## Results

### *A. fumigatus cdc43* mediates thermotolerance and polarized growth

To first examine the role of protein geranylgeranylation in *A. fumigatus* growth and virulence, we initiated this study by identifying the GGTase-I β-subunit. Mutation of this subunit is expected to cripple protein geranylgeranylation whereas farnesyltransferase activity remains intact. A BLAST search analysis utilizing the *S. cerevisiae* Cdc43p protein sequence performed through the *Aspergillus* genome database (aspergillusgenome.org) revealed a single homologous gene for the GGTase-I β-subunit, Afu6g06710 (29.9% amino acid identity), hereafter called *cdc43*. A *cdc43* deletion strain (Δ*cdc43*) was generated by replacement of the entire coding region in the uracil-auxotrophic Δ*akuB* strain with a selection cassette containing *A. parasiticus pyrG*. In order to confirm that phenotypic observations in the *cdc43* null mutants were due to gene loss, a complemented strain (Δ*cdc43:cdc43*) was achieved by ectopic integration of the *cdc43* coding locus, including 1 kb of upstream and 300 bp of downstream genomic sequence to function as native promoter and terminator. All manipulations were confirmed by PCR and Southern Blot analyses.

**Figure 1.**
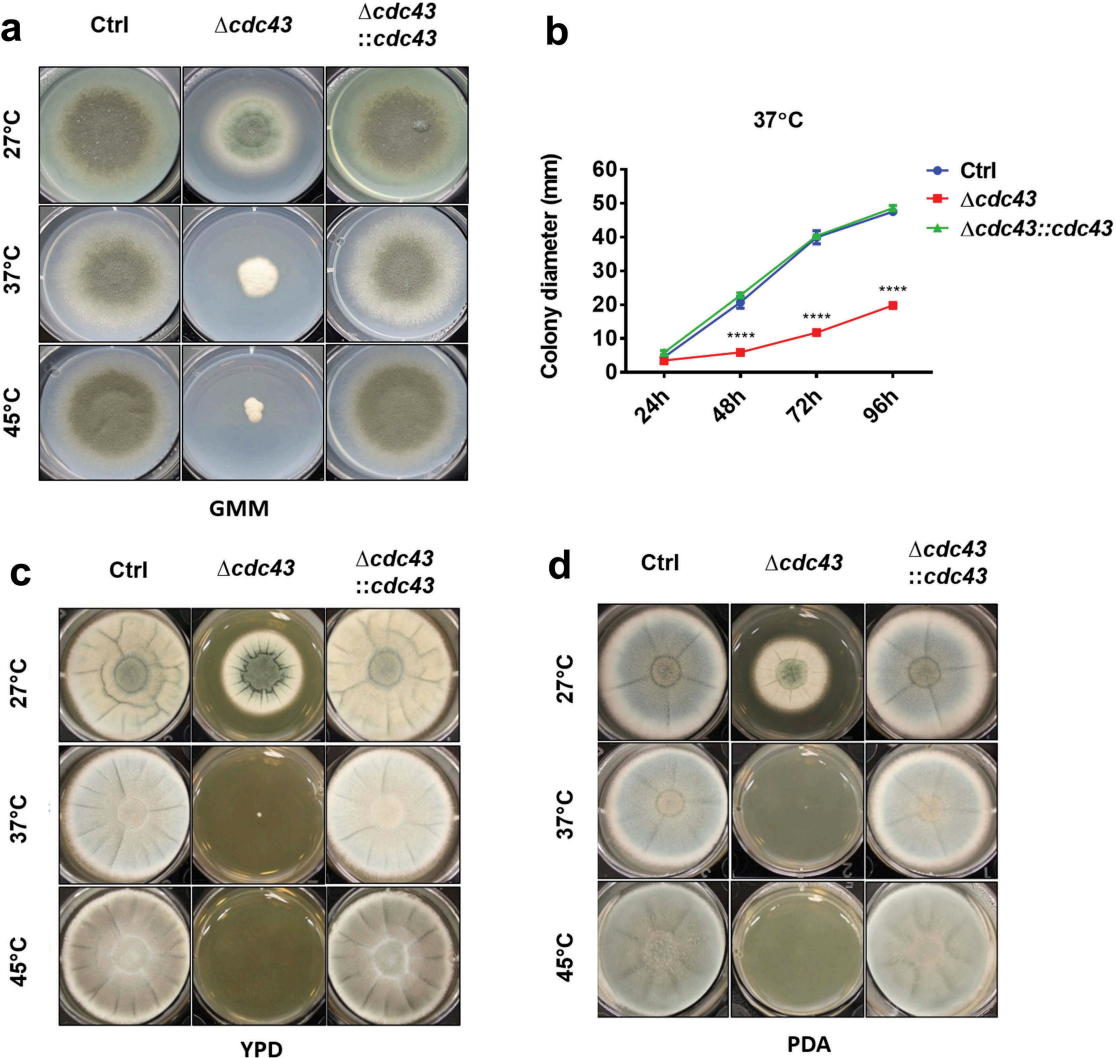
Protein geranylgeranylation promotes *A. fumigatus* thermotolerant growth. Conidia (5x10^3^) obtained from control (Ctrl), Δ*cdc43* or complemented (Δ*cdc43:cdc43*) strains were spot inoculated (5 µl) onto GMM (A), YPD (C), or PDA (D) agar. Growth and development of colony morphology at 27ºC, 37ºC, and 45ºC were monitored and images represent growth and colony development at the time that the control strain reached the plate boundary under each condition. Colony diameters (B) in GMM agar plates were measured every 24 h over 96 h of incubation at 37ºC. Mean and standard deviation (SD) of colonies derived from three independent mycelia cultures are represented. Asterisks indicate statistical differences in comparison with control strain (Ctrl) using two-way ANOVA with Tukey’s test for multiple comparison; ****p < 0.001.

The phenotypic characterization of the *A. fumigatus cdc43* null mutant was initiated by evaluating its ability to grow in defined minimal media at 27°C, 37°C and 45°C. Compared to the wild type and complemented strains, the Δ*cdc43* mutant displayed defective growth at all temperatures (Figure 1(a)). However, at 37°C and 45°C, the Δ*cdc43* mutant displayed increasingly reduced growth and loss of conidiation (Figure 1(a,b)). Culture of the mutant on rich media did not rescue growth rates at 27°C, suggesting nutritional deficiency was not underlying reduced growth of Δ*cdc43* (Figure 1(c,d)). In contrast, culture on rich media was found to exacerbate growth defects during culture at both 37°C and 45°C (Figure 1(c,d)).

**Figure 2.**
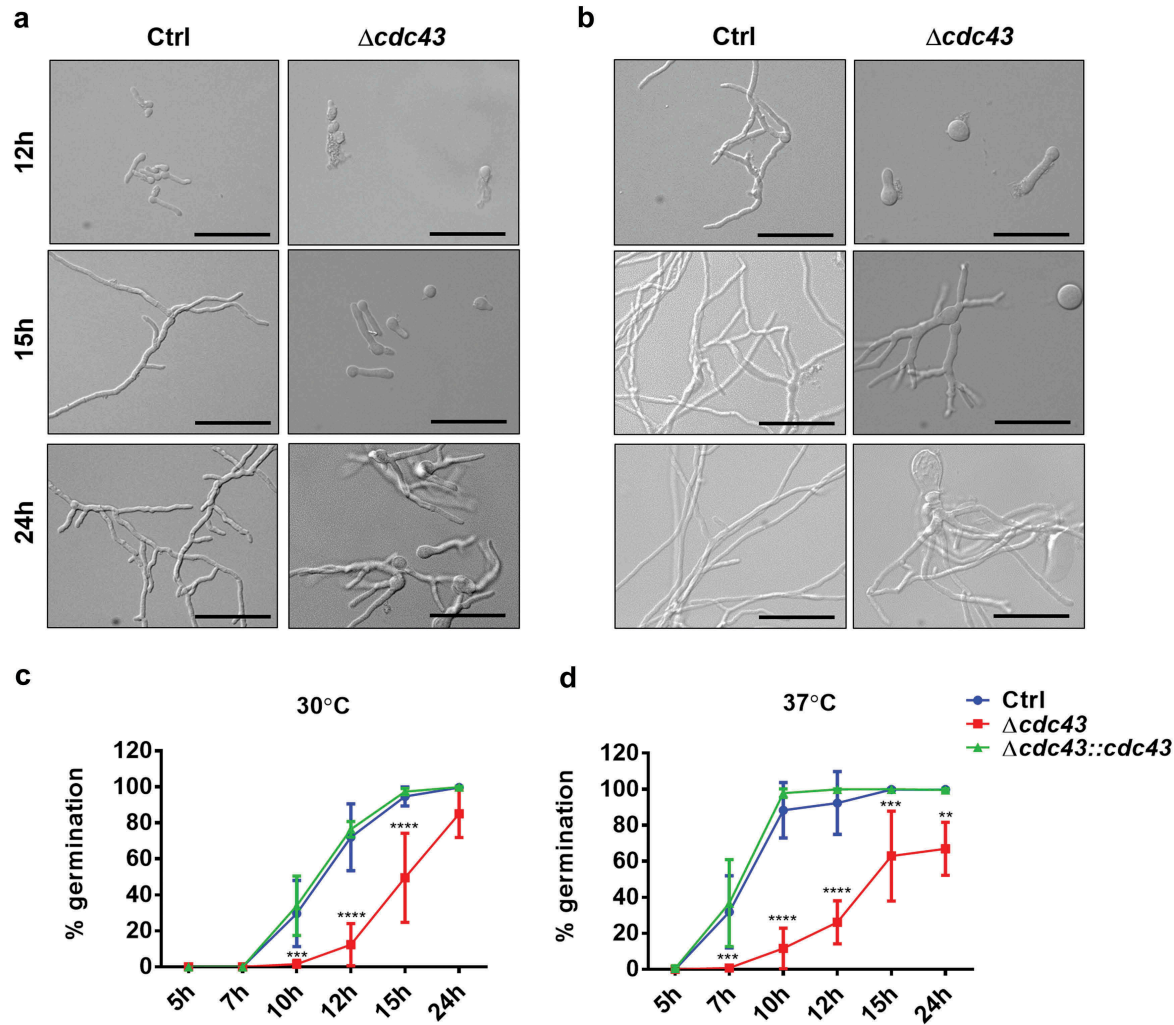
Lack of GGTase-I impairs *A. fumigatus* establishment of polarized growth. Conidia (10^5^) obtained from control (Ctrl), Δ*cdc43* or complemented (Δ*cdc43:cdc43*) strains were inoculated on sterile coverslips submerged in GMM broth (3 ml) in six-well plates. At the designated time points, coverslips were mounted onto slides and visualized by bright field microscopy. Micromorphology of germlings at 30ºC (A) and 37ºC (B) were evaluated after 12 h, 15 h, and 24 h (scale bar = 50 µm). Germ tube formation rates at 30ºC (C) and 37ºC (D) were determined by examining at least 100 conidia. Mean and standard deviation (SD) of five independent experiments are represented, and asterisks indicate statistical differences in comparison with control strain (Ctrl) using two-way ANOVA with Tukey’s test for multiple comparison; **p < 0.01, ***p < 0.001 and ****p < 0.0001.

To examine the effects of temperature on hyphal morphogenesis, micromorphology was analyzed in GMM broth at 30°C and 37°C. Conidia of the *cdc43* null mutant displayed delayed development of germ tubes accompanied by continuous isotropic swelling, resulting in unpolarized conidia with diameters measuring 2 times, at 30°C (Figure 2(a), 12 h), and 3 times, at 37°C (Figure 2(b), 12 h), the diameter of the control strain (Ctrl) swollen conidia. At 30°C, the Δ*cdc43* mutant formed wide, slow-growing germlings within 15 h of culture and eventually generated hyper-branched hyphae by 24 h (Figures 2(a), 15 h and 24 h). These phenotypes were further exacerbated at 37°C (Figures 2(b), 15 h and 24 h). Quantification of germ tube formation revealed an approximately 5-h delay in the Δ*cdc43* mutant at either temperature (Figure 2(c,d)). Moreover, by the end of 24 h of growth at 37°C, only 66.96% (±13.14%) of *cdc43* null mutant conidia were able to develop germ tubes (Figure 2(d)). The *cdc43* complemented strain (Δ*cdc43:cdc43*) was indistinguishable from the control strain in terms of radial growth and germination rates. These data suggest that protein geranylgeranylation mediates *A. fumigatus* thermotolerance and establishment of polarized growth.

### GGtase-I activity is important for *A. fumigatus* cell wall stress response and composition

**Figure 3.**
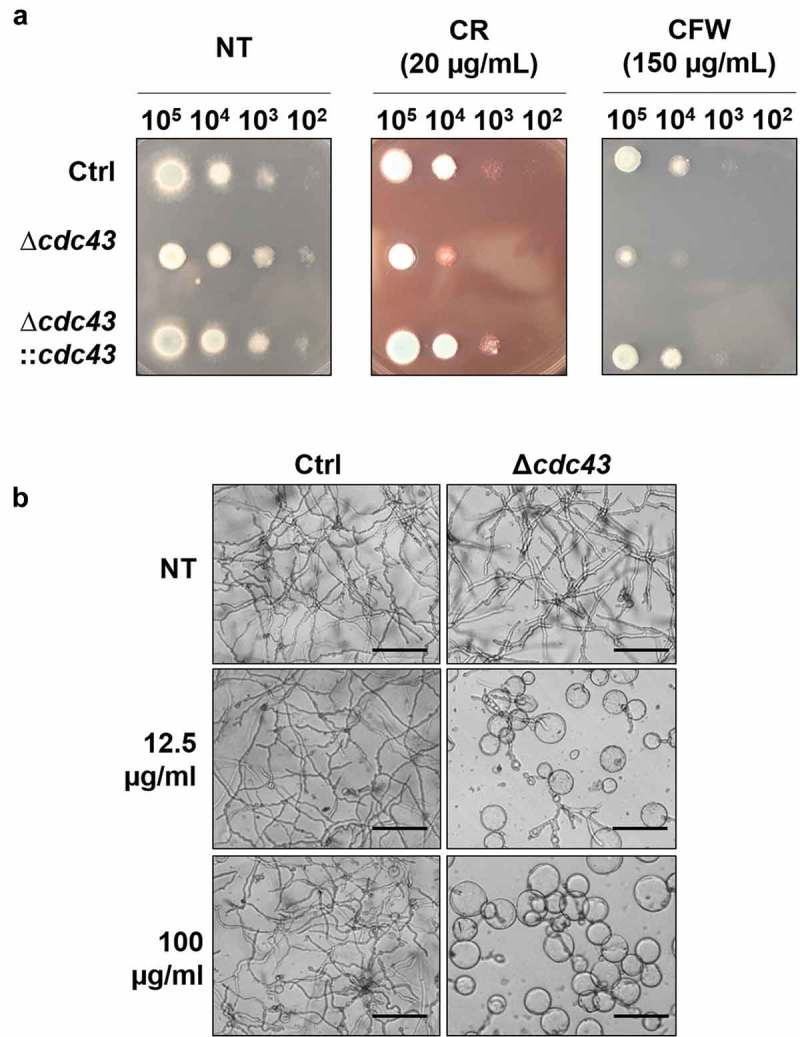
Loss of *cdc43* alters response to cell wall disrupting compounds. (A) Conidia (10^5^ to 10^2^) obtained from control (Ctrl), Δ*cdc43* or complemented (Δ*cdc43:cdc43*) strains (5 µl) were spot inoculated onto GMM agar plates impregnated with Congo Red (CR) or Calcofluor White (CFW). Colony size was examined after 48 h at 37ºC. (B) Micrographs of hyphae grown for 48 h at 37ºC in GMM broth with Nikkomycin Z (scale bar = 50 µm). Samples incubated in media with no compound (NT) were used as a control.

The ability of the *cdc43* isogenic set to respond to cell wall stress was evaluated by spot assay using GMM agar impregnated with the cell wall stress agents, Congo Red (CR) or Calcofluor White (CFW). We observed that Δ*cdc43* displayed increased sensitivity to both agents, indicated by loss of colony growth at lower inoculums (Figure 3(a)). Additionally, submerged culture in the presence of the chitin synthesis inhibitor Nikkomycin Z revealed dose-dependent inhibition of Δ*cdc43* growth at concentrations producing little-to-no effect in the control strain (Figure 3(b)). At the highest concentration of Nikkomycin Z, conidia of the Δc*dc43* mutant only grew isotropically as hyphal production was completely abolished (Figure 3(b)). Susceptibility to β-glucan synthesis inhibition by Caspofungin was also evaluated through a broth microdilution assay and revealed an increase in susceptibility by twofold dilutions (0.06 µg/ml) for Δ*cdc43* mutants in comparison to the control strain (0.25 µg/ml). Complementation of *cdc43* deletion recovered the ability to grow in the presence of CR and CFW (Figure 3(a)), and also recapitulated control strain growth in the presence of Nikkomycin Z and Caspofungin (data not shown).

**Figure 4.**
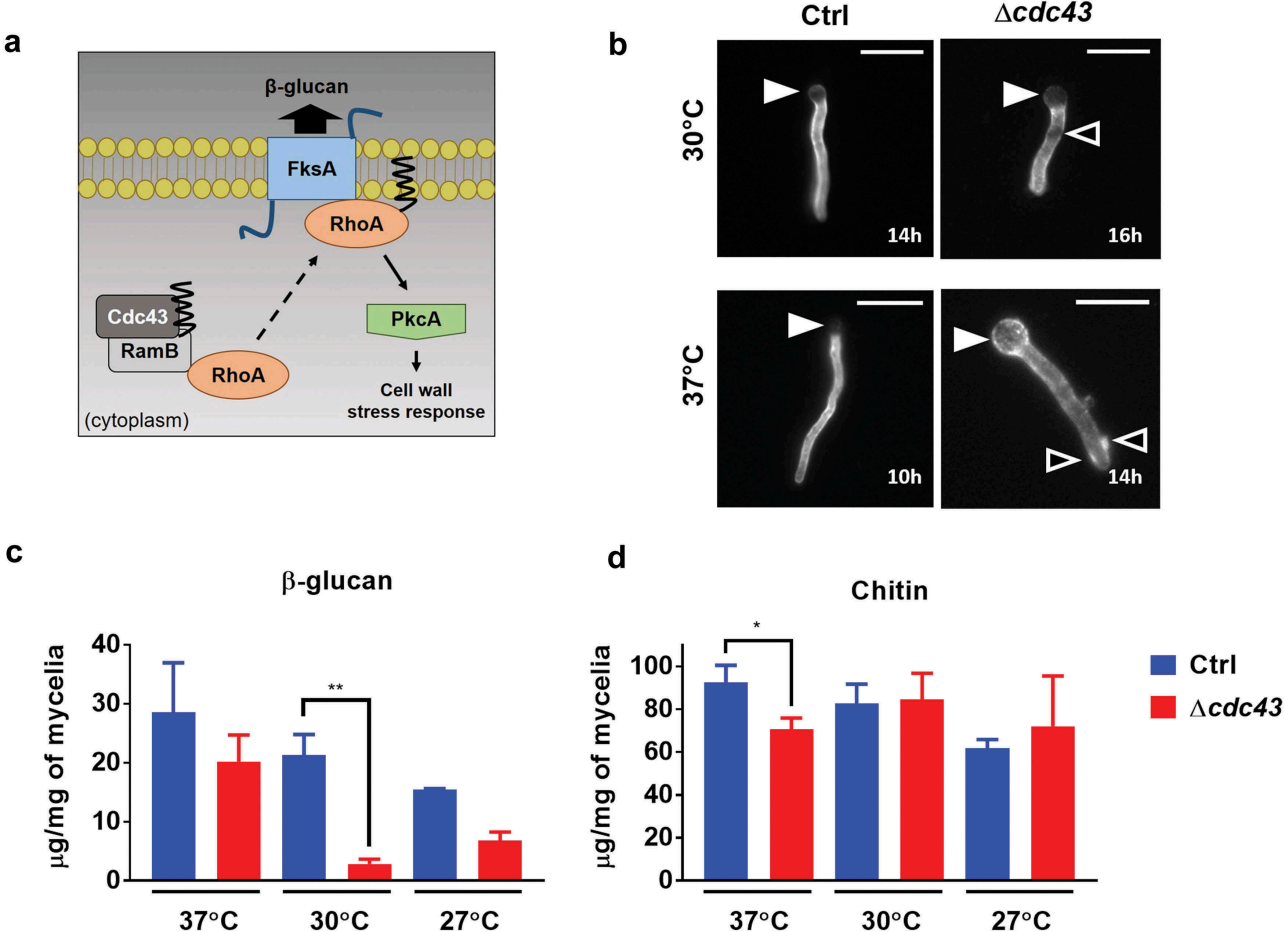
Protein geranylgeranylation is required for cell wall β-glucan composition. (A) The small GTPase, RhoA, is predicted to be prenylated by the GGTase-I complex (composed of the Cdc43-RamB heterodimer) based on analysis of CAAX motif composition [33]. Geranylgeranylation drives RhoA to cellular membranes where it functions as a regulatory subunit of the β-glucan synthase, FksA, complex [59] and activates downstream signaling cascades (via modulation of PkcA activity) to orchestrate cell wall stress responses [63]. (B) Conidia obtained from control (Ctrl) and Δ*cdc43* strains were inoculated in GMM broth and incubated at 30ºC or 37ºC at 250rpm. At designated time points, germlings were harvested and β-glucan staining was performed using a recombinant Fc-hDectin (10µg/ml) and anti-IgG-FITC (1:200). β-glucan staining of germlings was visualized by fluorescence microscopy (scale bar = 10 µm). Closed arrowheads denote the original germinating conidium and open arrowheads denote areas of patchy staining in hyphae of the Δ*cdc43* strain. Quantification of total β-glucan (C) and chitin (D) was performed after 18 h of incubation in GMM broth at 27°C, 30ºC or 37ºC with shaking at 250 rpm. Mean and standard deviation (SD) of samples derived from three independent mycelial cultures are represented, and asterisks indicate statistical differences in comparison with control strain (Ctrl) at the same temperature using unpaired t-test; *p < 0.05 and ****p < 0.0001.

A putative protein substrate of GGTase-I enzyme activity is RhoA, a small GTPase that plays a dual role in the activation of cell wall stress responses and in β-glucan biosynthesis (Figure 4(a)). Mislocalization of RhoA through the loss of GGTase-I activity would be predicted to result in reduced hyphal β-glucan content. Therefore, we evaluated cell wall distribution and composition by analyzing the localization and content of β-glucan and chitin in both the control and the Δ*cdc43* strains under non-stress culture conditions. Fluorescence staining of germlings for β-glucan visualization using a recombinant, soluble Dectin-1 probe revealed an irregular staining pattern of nascent germlings in the Δ*cdc43* strain (Figure 4(b)). Positive staining for β-glucan was also noted in the germinating conidium on the Δ*cdc43* mutant when grown at 37°C, whereas the original conidium was nearly devoid of β-glucan signal in the control strain at 37°C and in either strain at 30°C (Figure 4(a)). These findings suggest that deposition of β-glucan in the cell wall is altered in the Δ*cdc43* mutant, especially during growth at higher temperatures. In contrast, fluorescence staining for chitin distribution using calcofluor white revealed no alteration of localization of this polysaccharide (data not shown). Total β-glucan quantification in hyphae grown at 37°C, 30°C and 27°C revealed significantly lower amounts of this polysaccharide in the *cdc43* null mutant at 30°C (Figure 4(c)). Although β-glucan content trended lower at 27°C and 37°C the differences were not statistically significant due to assay variability (Figure 4(c)). In contrast, total chitin quantification did not show significant differences among the strains at lower temperatures and only produced significantly different results at 37°C (Figure 4(d)). The impaired β-glucan composition of the cell wall and the increased susceptibility to cell wall perturbing agents suggest that protein geranylgeranylation is important for substrate proteins to function in *A. fumigatus* cell wall biosynthesis, assembly and/or stress response.

### GGtase-I activity is not required for *A. fumigatus* virulence in experimental invasive aspergillosis

**Figure 5.**
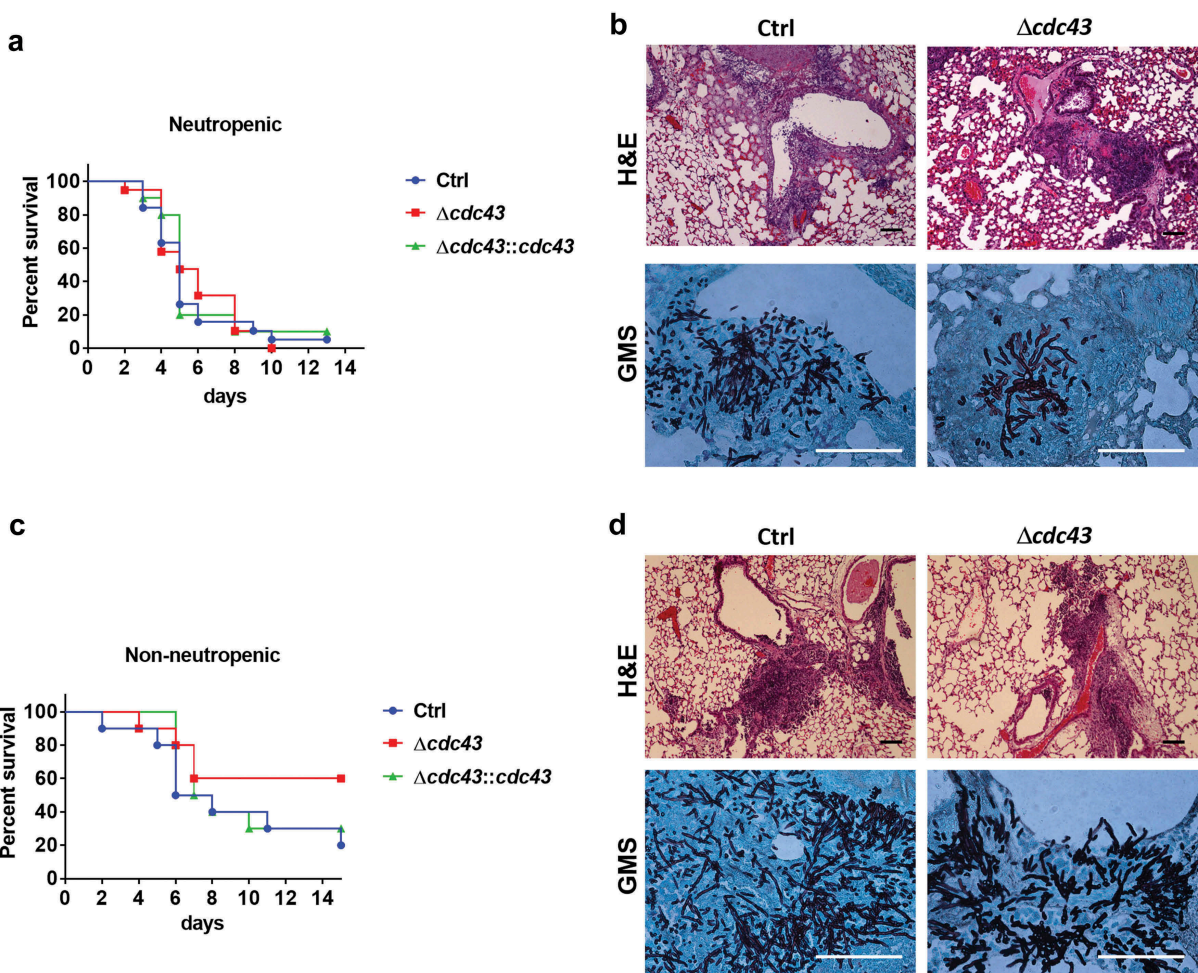
The Δ*cdc43* mutant retains virulence during experimental invasive aspergillosis. Neutropenic (A, B) and non-neutropenic (C, D) CF-1 female mice were intranasally inoculated with 10^5^ conidia obtained from control (Ctrl), Δ*cdc43* or complemented (Δ*cdc43:cdc43*) strains. Survival was monitored for two weeks (A, C) and analyzed using a Log-rank (Mantel-Cox) test. Histology sections of lung tissue stained with hematoxylin and eosin (H&E) and Gomori methenamine silver (GMS) collected 3 days after infection (scale bars = 100 µm) (B, D).

Since Cdc43 plays a crucial role in *A. fumigatus* growth at physiologically relevant temperatures *in vitro*, and in cell wall composition, we hypothesized that deletion of *cdc43* would have a negative impact on pathogenesis during a murine model of invasive aspergillosis. Surprisingly, virulence was not attenuated during infection with the Δ*cdc43* mutants in both neutropenic (Figure 5(a,b)) and non-neutropenic mice (Figure 5(c,d)). Infected mice displayed similar survival rates in the neutropenic model, reaching 50% of mortality around 5 (Ctrl and Δ*cdc43::cdc43*) and 6 days (Δ*cdc43*) post infection (Figure 5(a)). In the non-neutropenic model, 60% of the mice did not succumb to Δ*cdc43* infection in comparison to 20% of survival in the control strain, but these rates were not sufficient to establish a statistical difference (Figure 5(c)). Histological analysis of infected lungs in both immunosuppression models demonstrated hyphal growth around bronchioles and tissue invasion within 3 days of infection with all strains (Figure 5(b,d)). Importantly, although hyphae appeared to be stunted, the hyper-branching phenotype previously observed *in vitro* was not detected in the lung tissue of mice infected with Δ*cdc43* (Figure 5(b,d) and data not shown).

### The shared prenyltransferase α-subunit, RamB, is essential for A. fumigatus growth and virulence

**Figure 6.**
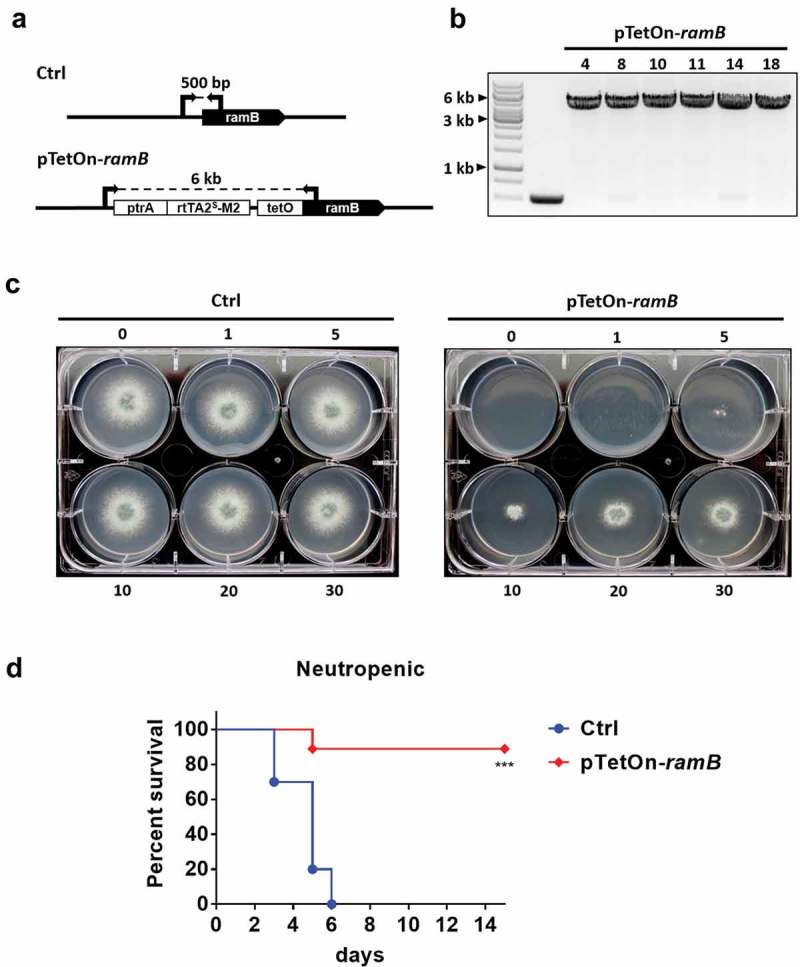
Protein prenylation is essential for *A. fumigatus* viability and virulence. (A) Schematic representation of pTetOn promoter replacement for *ramB*. The pTetOn cassette was previously described and contains the pyrithiamine (ptrA) resistance marker [39]. Arrows indicate areas where screening primers anneal to the genomic template. (B) Homologous integration of the pTetOn cassette was confirmed by PCR. (C) pTetOn-*ramB* growth in GMM media at 37°C was dependent on the presence of doxycycline in the growth media. 10^4^ conidia from each strain were spot inoculated onto GMM with the indicated concentrations of doxycycline and incubated at 37°C for 48 h. Numbers on each well indicate concentration (µg/ml) of doxycycline. (D) Neutropenic CF-1 female mice were intranasally inoculated with 10^5^ conidia obtained from control (Ctrl) or the tetracycline-inducible *ramB* (pTetOn-*ramB*) strains. Survival was monitored along 14 days and analyzed using Log-rank (Mantel-Cox) test. Asterisks indicate a statistically significant difference in comparison with the control group (Ctrl); ***p < 0.001.

Our results showed that lack of GGTase-I activity impacts *A. fumigatus* growth, morphology and cell wall composition, but not virulence. In contrast, we previously reported that mutants lacking the β-subunit of FTase presented attenuated virulence, in addition to impaired growth rates, decreased conidial viability and abnormal distribution of nuclei during polarized growth [33]. Both prenyltransferases share a common α-subunit, RamB, that is predicted to stabilize the dimeric complex and participate in the catalytic reaction transferring prenyl moieties to substrate proteins [3]. We hypothesized that complete lack of cellular prenylation, imparted by deletion of the α-subunit, would be deleterious in this fungal pathogen. Attempts to create a *ramB* null mutant were unsuccessful. Therefore, we generated a strain with inducible expression of *ramB* (pTetOn-*ramB*) by replacing the native promoter with a tetracycline-inducible promoter (Figure 6(a,b)). Expression of *ramB* in this strain can be titrated by the addition of increasing amounts of doxycycline to the culture media. After culturing the pTetOn-*ramB* mutant in GMM media with increasing concentrations of doxycycline (0 to 30 µg/ml), we observed that this strain was completely unable to grow in concentrations lower than 5 µg/ml whereas the control strain was unaffected at all concentrations tested (Figure 6(c)). These data support that *ramB* is an essential gene during *A. fumigatus in vitro* growth.

In order to evaluate the importance of *ramB* expression during invasive growth *in vivo*, we infected mice with the control and pTetOn-*ramB* strains in both neutropenic and non-neutropenic mouse models. The pTetOn-*ramB* mutant displayed markedly attenuated virulence in the neutropenic (Figure 6(d)) model and was completely avirulent in the non-neutropenic model of invasive aspergillosis (Supplemental Figure 2A). Histological analyses did not reveal the presence of hyphae or tissue damage in the lungs within 3 days of infection with the pTetOn-*ramB* mutant in both immunosuppression models (Supplemental Figure 2B). Therefore, we conclude that *ramB* is essential for growth *in vitro* and to cause invasive aspergillosis in mice.

### Both α- and β-subunits of GGTase-I are required for C. albicans virulence in murine model of disseminated infection

**Figure 7.**
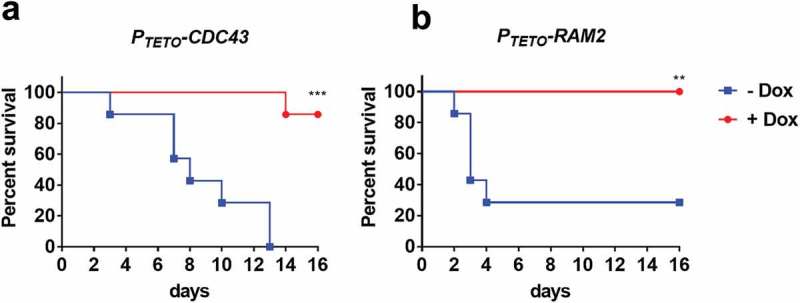
Both *CDC43* and *RAM2* are essential for *Candida albicans* virulence in a mouse model of disseminated infection. Female BALB/c mice (6–8 week old) were randomly assigned into two treatment groups, one provided a gel food formulation supplemented with doxycycline (2 mg/ml) (+ Dox), the second gel food alone (- Dox). Half the mice in either group were then inoculated with 5 × 10^5^ yeast cells of *C. albicans* strain *P_TETO_-CDC43* (A), and the other half with strain *P_TETO_-RAM2* (B) via tail vein injection (n = 7 per experimental group). Mice were monitored for 16 days, and those showing signs of distress humanely euthanized. Survival of mice in either treatment group was then compared using the Log-rank (Mantel-Cox) test, and asterisks indicate a statistically significant difference among the groups; **p < 0.01 and ***p < 0.001.

Our studies with *A. fumigatus* established that *ramB*, but not *cdc43*, was essential for virulence. Similarly, the shared prenyltransferase α-subunit homolog, *RAM2*, was also found to be required for *C. glabrata* to infect mice [27]. In *C. albicans*, the α-subunit of prenyltransferase is essential for viability [21], whereas lack of *CDC43* has been shown to affect cell morphology and filamentous growth *in vitro* [31]. However, the importance of prenyltransferase activity for *C. albicans* to cause disease within its mammalian host has not yet been determined. We therefore evaluated the virulence of two *C. albicans* strains, one with tetracycline-repressible expression of *CDC43* (*P_TETO_-CDC43*), and the other with tetracycline-repressible expression of *RAM2* (*P_TETO_-RAM2*) [40], in a murine model of hematogenously disseminated candidiasis. The essentiality of *RAM2* (but not *CDC43*), was supported by the fact that the *P_TETO_-RAM2* strain was unable to grow on YPD + 5 µg/ml doxycycline agar (data not shown). BALB/c mice were next assigned to one of two treatment groups, one provided a gel food supplemented with doxycycline, the other provided gel food alone. After 3 days, half the mice in each treatment group were infected with *P_TETO_-CDC43*, and the other half with *P_TETO_-RAM2*, via the lateral tail vein (n = 7 per experimental group). In the absence of doxycycline, all 7 mice infected with *P_TETO_-CDC43*, and 5 of 7 infected with the *P_TETO_-RAM2* strain succumbed to infection (Figure 7(a,b)). However, only 1 of 7 *P_TETO_-CDC43* infected mice, and none of the *P_TETO_-RAM2* infected mice succumbed to infection in the doxycycline treatment group. These findings indicate that the expression of both *CDC43* and *RAM2* is required for *C. albicans* pathogenesis.

## Discussion

The contributions of protein prenylation in eukaryotic cell biology are continuously under investigation, and the demonstration that CaaaX-residues can also be prenylated [4] expanded even more the repertoire of possible prenylated proteins. Prenylation controls the localization of several proteins that are crucial for the viability of fungal pathogens. *In silico* analyses revealed that *A. fumigatus* encodes 28 putative prenylated CaaX proteins [33]. Among them are important regulators of cell growth and development, including small GTPases such as Ras- and Rho- family proteins. We had previously shown that protein farnesylation promotes *A. fumigatus* growth, nuclear distribution, conidial viability and virulence [33]. In this study, we analyzed the contribution of protein prenylation in general, and specifically protein geranylgeranylation, to the growth and virulence of *A. fumigatus*.

We started by creating and characterizing a strain that lacked the *cdc43* gene, that encodes the β-subunit of geranylgeranyltransferase type-I. Since *A. fumigatus* is a thermotolerant organism [44,45], we analyzed if the absence of GGTase-I would have an impact on the ability of this pathogen to grow at host physiological, or greater, temperatures. The Δ*cdc43* mutant presented reduced hyphal growth and thermo-sensitivity, especially while growing in rich media at 45°C. Impaired ability to grow at 37°C was also observed after disruption of *RAM1* in *S. cerevisiae* [26] and *RAM1* [28] and *CDC43* [32] in *C. neoformans*. In fact, *RAS1*, a farnesylated protein, has been shown to directly control *C. neoformans* thermotolerant growth [46]. We speculate that protein geranylgeranylation is crucial for *A. fumigatus* heat shock response, especially under conditions where metabolic activity is intensified, such as when resources are abundant. We compared the list of *A. fumigatus* putative prenylated proteins [33] with a list of proteins that were found regulated by Albrecht *et. al*. (2010) during a temperature shift from 30°C to 48°C [45], however, we found no matches. *A. fumigatus* null mutants for *rho2* and *rho4* were found sensitive to growth at 48°C [47], and these proteins are potentially prenylated. It is likely, therefore, that the cell wall and temperature-sensitive phenotypes are directly linked. Two other genes identified as mediators of thermotolerance in *A. fumigatus, cgrA* [48] and *thtA* [49] do not have a predicted CaaX motif. Although this suggests that these proteins are not likely to be directly impacted by GGTase-I activity, we cannot rule out that they may be indirectly associated with the thermosensitive phenotype observed here.

We observed that lack of GGTase-I had an impact on fungal cell morphology. The Δ*cdc43* mutant presented larger swollen conidia as well as wide, hyper-branched hyphae. These phenotypes were exacerbated during growth at 37°C. In a similar way, *C. neoformans* [32] and *S. cerevisiae CDC43* mutants presented altered morphology, such as failure to bud and continuous cell growth at 37°C, what suggests abnormal control of cell cytokinesis. Accordingly, *S. cerevisiae* mutants for *CDC42* [50], which is a substrate for GGTase-I, present similar altered morphology. *A. fumigatus* has a *CDC42* homolog that is a putative substrate of GGTase-I [33], but this gene has not yet been studied. *CDC42* is involved in the morphogenesis of *S. cerevisiae* [51], *C. albicans* [52], *C. neoformans* [53], *A. niger* [54] and *A. nidulans* [55,56]. In addition, RacA is also a potentially prenylated protein, and mediates polarized growth in *A. fumigatus* [57]. As with the Δ*cdc43* strain, *racA* null mutants present impaired ability to conidiate in GMM [57,58]. The lack of this protein in *A. niger* produces hyper-branching hyphae, and double deletion of *racA* and *cdc42* leads to malformed germlings during growth in GMM [54]. Taking this into consideration, the *A. fumigatus* homolog for *CDC42* and RacA are likely substrate proteins that have altered function by the absence of GGTase-I activity in the Δ*cdc43* mutant.

The disruption of *cdc43* created mutants hypersensitive to cell wall disrupting agents, namely Congo Red, Calcofluor White, Nikkomycin Z, and Caspofungin, indicating defects in cell wall biosynthesis, assembly and/or integrity. In fact, analysis of cell wall β-glucan by both fluorescence microscopy and quantification using the aniline blue assay revealed the altered distribution and reduced amount of this polysaccharide in the Δ*cdc43* strain. β-glucan is synthesized from glucose by the β-1,3-glucan synthase complex, constituted by the catalytic subunit FksA, and the regulatory subunit RhoA [59–61]. In *S. cerevisiae*, prenylation of Rho1p is required for the activation of β-1,3-glucan synthase [61]. We predict that RhoA is at least partially mislocalized in the Δ*cdc43* mutant which, in turn, impairs cell wall β-glucan production by inhibiting activation of the β-1,3-glucan synthase complex. *A. fumigatus rho2* and *rho4* null mutants also display sensitivity to CR and CFW [47]. The hypersensitivity of the Δ*cdc43* mutant to Caspofungin may further suggest that FksA activity is reduced potentially as a result of RhoA mislocalization. Also, among the possible prenylated proteins in *A. fumigatus* are two uncharacterized genes [33] that have homology to a *S. cerevisiae* and *C. albicans* chitin-synthase regulator (*CHR4*). In these fungal species, lack of *CHS4* reduces cell wall chitin content. However, we did not identify changes in chitin levels after visualization of hyphae stained with CFW by fluoresce microscopy, nor by quantification of glucosamine-equivalents in hyphal mats. Even though chitin content is normal, we did note hypersensitivity of Δ*cdc43* to chitin synthesis inhibition by Nikkomycin Z. A possible explanation is that, since β-glucan content is impaired in this mutant, chitin is the major component stabilizing the cell wall such that inhibition of chitin synthesis by Nikkomycin Z became completely deleterious to polarized morphogenesis. In addition, the predicted GGTase-I substrates RhoA, Rho2, and Rho4 are known to participate in the cell wall integrity signaling, which is crucial to counter stress agents such as CFW, CR and Caspofungin [47]. Further studies defining prenyltransferase substrate specificity and Rho protein localization in the Δ*cdc43* background will be required to pursue this possibility.

After characterizing the importance of protein geranylgeranylation in *A. fumigatus*, we sought to evaluate the impact of protein prenylation in general. The *ramB* gene encodes the α-subunit of both FTase and GGTase-I. Lack of RamB is expected to completely abolish CaaX protein prenylation and cause the concurrent mislocalization of all substrate proteins. Accordingly, we were unable to delete *ramB*. Replacement of the native promoter with a tetracycline inducible promoter (TetOn) generated a strain completely dependent on the presence of doxycycline to survive. These findings are in accordance with other studies showing that *ramB* homologs are essential for *S. cerevisiae* [26], *C. albicans* [21] and *C. glabrata* [27] viability. We assume that inhibition of *ramB* expression severely impacts pathways that depend on protein prenylation, such as signal transduction mediated by Ras and Rho-proteins. Lack of prenylation likely affects a range of essential cellular processes, such as growth, differentiation and establishment of polarity.

The temperature sensitive phenotype and the inability to maintain proper polarity indicated that the *A. fumigatus* Δ*cdc43* mutant would likely present attenuated virulence. Surprisingly, our virulence studies suggested that complete lack of GGTase-I activity did not affect *A. fumigatus* ability to grow in the host environment. The same was observed in *thtA* mutants and six other mutants that presented normal growth at 25°C, but failed to grow at 48°C, and presented no reduction in virulence in the murine model of invasive aspergillosis [49]. In addition, an *A. fumigatus racA* deletion strain was as virulent as Af293 in both neutropenic and non-neutropenic models of invasive aspergillosis, despite loss of polarity and impaired growth phenotypes *in vitro* [57]. In fact, little-to-no morphological changes were identified in the Δ*cdc43* hyphae growing in the lung tissue compared to the control strain. These data could indicate that, once inside the mammalian host, *A. fumigatus* has a reduced requirement for protein geranylgeranylation. However, we cannot currently rule out the possibility that the altered cell wall of the Δ*cdc43* mutant leads to differences in the immune response that impact this outcome.

*CDC43* was previously described as a non-essential gene in *C. albicans*, although null mutants displayed abnormal morphology and decreased the ability to form hyphae [31]. In this study, using a tetracycline-repressible strain, we demonstrated that suppression of *C. albicans CDC43* expression resulted in increased survival of mice in a murine model of invasive candidiasis. These data establish that whereas geranylgeranylation is not required for *A. fumigatus* virulence, it is crucial for *C. albicans* virulence. We also demonstrated that the α-subunit of both FTase and GGTase-I is crucial for fungal growth and ability to infect the host. The *A. fumigatus* pTetOn-*ramB* strain presented no virulence in both neutropenic and non-neutropenic models of invasive aspergillosis. Similarly, suppression of *RAM2* expression is sufficient to render *C. albicans* avirulent, establishing that prenylation is essential for pathogenicity.

The data reported here supports the exploration of fungal protein prenylation as a target for antifungal drug development. *In vitro* effectiveness of some prenyltransferase inhibitors have been investigated, and revealed antifungal activity against *C. neoformans* [23,24], *Aspergillus* spp. and *Candida* spp. [18]. Additional reports indicate that these inhibitors are not active against the whole fungal cell, although they are able to inhibit purified prenyltransferases. These discrepant results are possibly due to the inability of the tested compounds to enter the fungal cell or may result from potential cross-prenylation of substrate proteins due to promiscuous substrate specificity [9,21,31,62]. To the best of our knowledge, no *in vivo* studies analyzing antifungal efficacy of protein prenyltransferase inhibitors have been conducted. Virulence studies performed with prenyltransferase mutants demonstrate their importance for *in vivo* growth of fungal pathogens. In *C. neoformans* [28] and *A. fumigatus* [33], lack of farnesyltransferase activity diminishes virulence. In addition, mice infected with *C. glabrata* mutants that harbor a tetracycline-repressible *RAM2* display lower numbers of viable fungal cells in the kidneys when treated with doxycycline [27]. The fact that the α-subunit of the CaaX prenyltransferases is crucial for pathogenicity of *C. albicans, C. glabrata*, and *A. fumigatus* is encouraging. Although a well-conserved pathway in both mammalian and fungal cells, important structural differences [6,17,24] raise the possibility of specifically targeting the fungal enzymes, avoiding undesired side effects during the antifungal therapy. Simultaneous inhibition of FTase and GGTase-I activity or the impairment of the dimerization of the α-subunit with the β-subunits of prenyltransferases seem to be a promising strategy to combat fungal infections in a broader spectrum. Therefore, further investigations in the development or repurposing of inhibitors of prenylation will be valuable in the fight against invasive fungal diseases.
