# Supplementary material for: Differential requirements of protein geranylgeranylation for the virulence of human pathogenic fungi
Source: Virulence. 2019 May 25;10(1):511–26. doi: 10.1080/21505594.2019.1620063 (PMC6550545; doi:10.1080/21505594.2019.1620063)
Supplement: Supplemental Material [file kvir-10-01-1620063-s001.zip › Supplemental Fig caption.docx]

**Supplemental Figure 1. Models of the genetic manipulations in *cdc43* and *ramB* of *A. fumigatus*.** The *cdc43* deletion mutant (Δ*cdc43*) was obtained by replacement of the entire ∆*akuB* *cdc43* coding sequence using the *A. parasiticus* *pyrG* gene (A). Complementation of Δ*cdc43* (Δ*cdc43*::*cdc43*) was achieved by the ectopic integration of the entire *cdc43* coding sequence with a hygromycin resistance cassette (B). The *ramB* (pTetOn-*ramB*) tetracycline-inducible strain was developed by replacement of the native *ramB* promoter with the pyrithiamine-based Tet-On cassette (C).

**Supplemental Figure 2. *ramB* expression is crucial for *A. fumigatus* virulence during experimental invasive aspergillosis.** CF-1 female mice were intranasally inoculated with 10^5^ conidia obtained from control (Ctrl) or the tetracycline-inducible *ramB* (pTetOn-*ramB*) strains. (A) Non-neutropenic mice survival was monitored along 14 days and analyzed using Log-rank (Mantel-Cox) test. Asterisks indicate a statistically significant difference in comparison with the control group (Ctrl); ***p<0.001. (B) Histology sections of lung tissue stained with hematoxylin and eosin (H&E) and Gomori methenamine silver (GMS) collected 3 days after infection (scale bars = 100µm) (B, D).
