# Supplementary figures and images for: Differential requirements of protein geranylgeranylation for the virulence of human pathogenic fungi
Source: Virulence. 2019 May 25;10(1):511–26. doi: 10.1080/21505594.2019.1620063 (PMC6550545; doi:10.1080/21505594.2019.1620063)

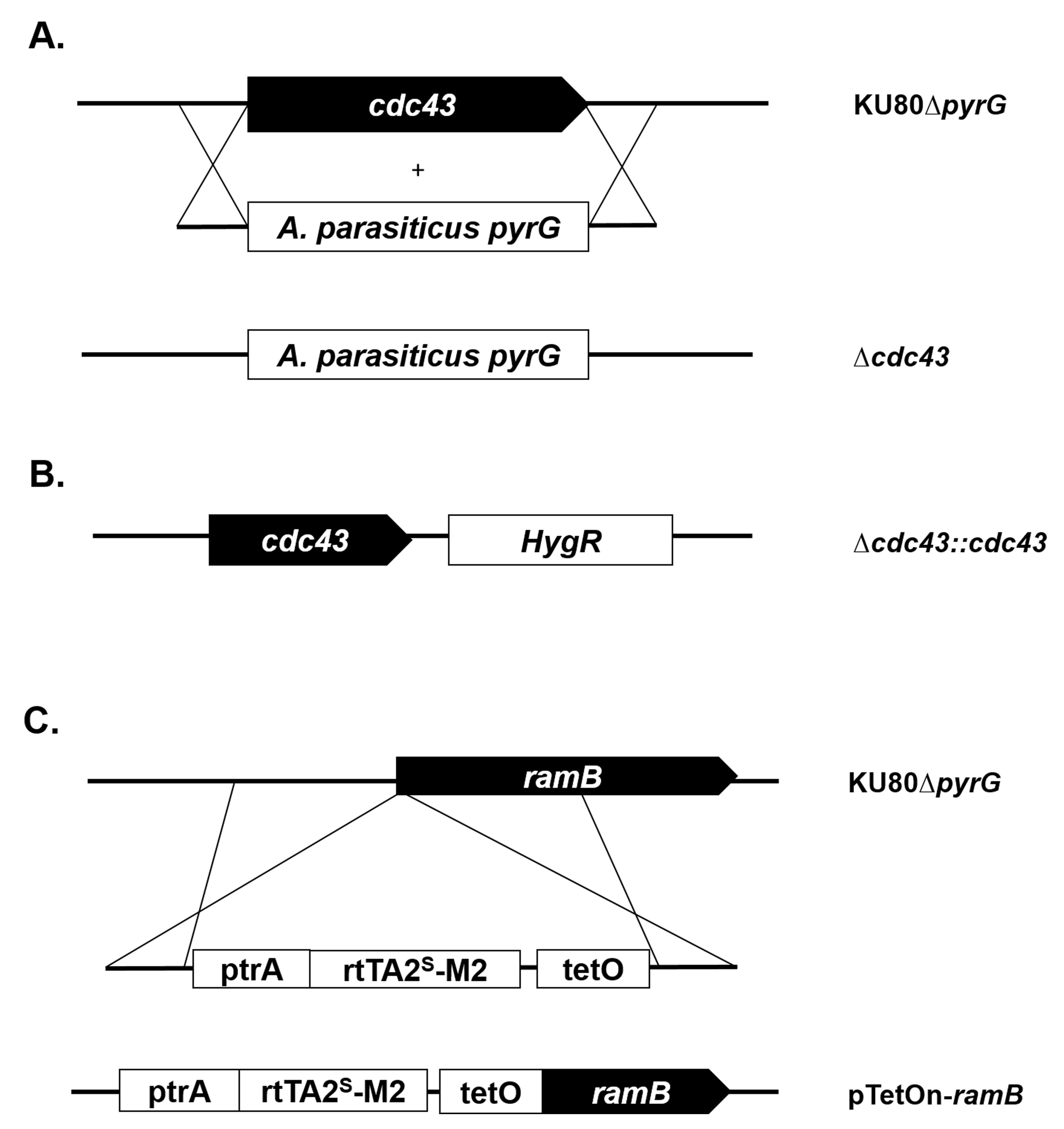

Supplement: Supplemental Material [file kvir-10-01-1620063-s001.zip › Sup Fig 1.jpg]

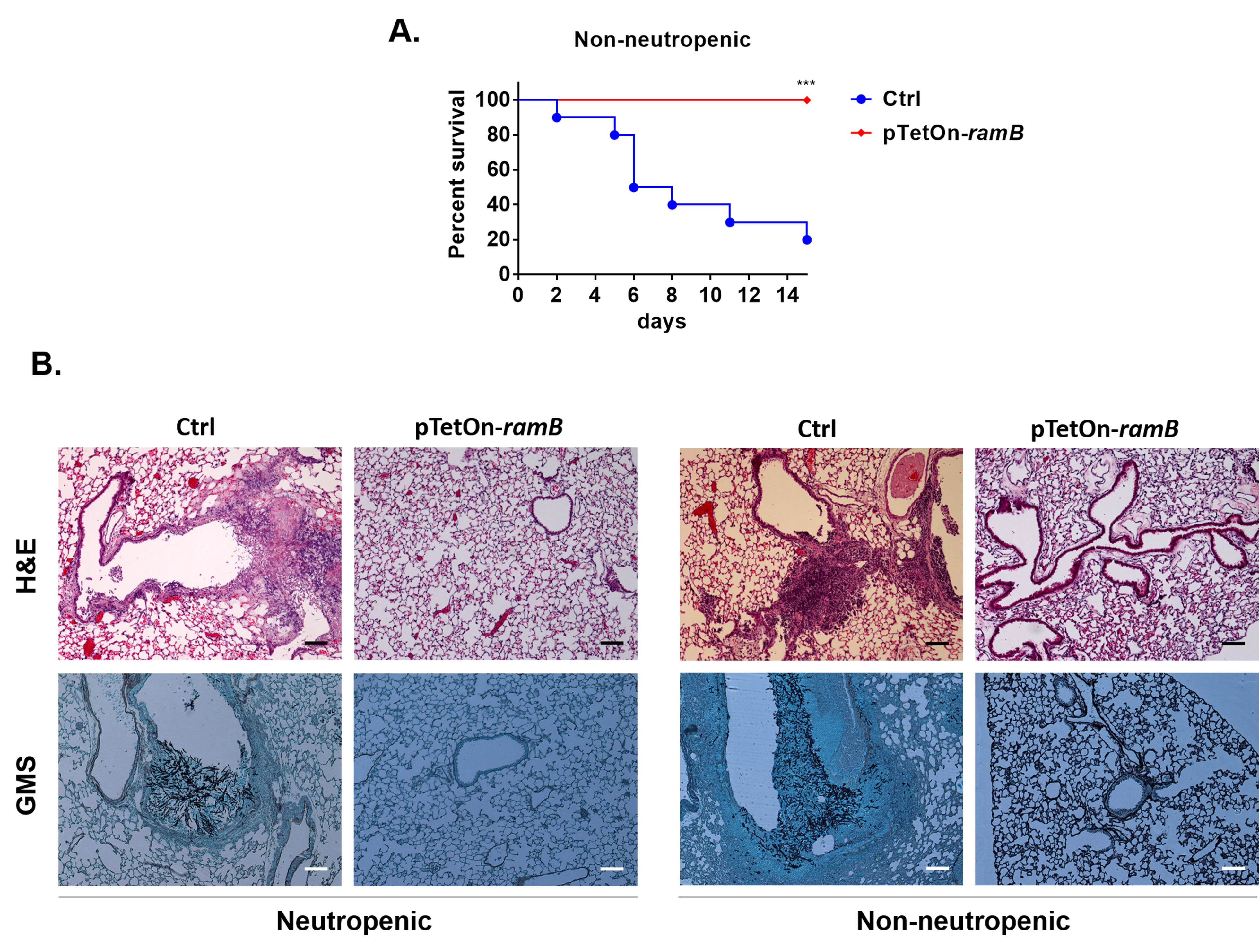

Supplement: Supplemental Material [file kvir-10-01-1620063-s001.zip › Sup Fig 2.jpg]
